# Potential role of *Plasmodium falciparum*-derived ammonia in the pathogenesis of cerebral malaria

**DOI:** 10.3389/fnins.2015.00234

**Published:** 2015-07-03

**Authors:** Sammy Kimoloi, Khalid Rashid

**Affiliations:** ^1^Department of Medical Laboratory Sciences, Masinde Muliro University of Science and TechnologyKakamega, Kenya; ^2^Biochemistry and Molecular Biology Department, Egerton UniversityNakuru, Kenya

**Keywords:** *P. falciparum*, cerebral malaria, parasite-derived ammonia, elevated ammonia, pathogenesis

## Abstract

Cerebral malaria (CM) is the most severe complication associated with *Plasmodium falciparum* infection. The exact pathogenic mechanisms leading to the development of CM remains poorly understood while the mortality rates remain high. Several potential mechanisms including mechanical obstruction of brain microvasculature, inflammation, oxidative stress, cerebral energy defects, and hemostatic dysfunction have been suggested to play a role in CM pathogenesis. However, these proposed mechanisms, even when considered together, do not fully explain the pathogenesis and clinicopathological features of human CM. This necessitates consideration of alternative pathogenic mechanisms. *P. falciparum* generates substantial amounts of ammonia as a catabolic by-product, but lacks detoxification mechanisms. Whether this parasite-derived ammonia plays a pathogenic role in CM is presently unknown, despite its potential to cause localized brain ammonia elevation and subsequent neurotoxic effects. This article therefore, explores and proposes a potential role of parasite-derived ammonia in the pathogenesis and neuropathology of CM. A consideration of parasite-derived ammonia as a factor in CM pathogenesis provides plausible explanations of the various features observed in CM patients including how a largely intravascular parasite can cause neuronal dysfunction. It also provides a framework for rational development and testing of novel drugs targeting the parasite's ammonia handling.

## Introduction

Cerebral malaria (CM) is one of the most common and fatal complications associated with *Plasmodium falciparum* infection (Newton et al., 2000). Despite the availability of effective anti-malarial drugs, CM mortality rates in malaria afflicted regions remain high, ranging between 15 and 20% (Mishra and Newton, 2009). This has partly been attributed to the fact that a number of patients succumb to CM before the onset of administered anti-malarial therapeutic effects (Mishra and Newton, 2009). Transient and persistent neurocognitive impairments are also common among cerebral malaria survivors, mostly children (van der Wal et al., 2005; Idro et al., 2010). Several mechanisms have been hypothesized to play a role in the pathogenesis of CM including; mechanical obstruction of brain microvasculature by sequestered parasitized red blood cells (PRBCs), inflammation, hemostatic dysfunction, excessive parasite-derived lactate, and oxidative stress (Berendt et al., 1994; Medana et al., 2001; van der Heyde et al., 2006; Mariga et al., 2014). However, the exact pathogenesis of CM remains poorly understood and the various clinicopahtological features of human CM cannot be fully explained by the existing hypotheses (van der Heyde et al., 2006). Additionally, adjunctive therapies targeting some of these earlier proposed pathogenic mechanisms have failed to improve CM treatment outcomes or have been found detrimental in human clinical studies (White et al., 2013). Exploration of other potential pathogenic factors is therefore needed in order to understand the underlying CM pathogenesis and develop adjunctive therapies that can possibly prolong anti-malarial therapeutic window and reduce neurocognitive sequelae among survivors.

Ammonia is a neurotoxic metabolic by-product whose blood concentration is tightly maintained at levels of less than 50 μmol/L mainly by the action of liver glutamine synthetase, which has a lower Km for ammonia than carbamoyl-phosphate synthetase (Arn et al., 1990; Cohn and Roth, 2004; Adeva et al., 2012). The brain astrocytic glutamine synthetase also plays a minor role, converting upto 25% of blood derived ammonia to glutamine (Eid and Lee, 2013). Several factors including, acute liver failure (ALF), urea cycle disorders, Reye's syndrome and hepatic drug toxity may however interfere with the normal ammonia metabolism leading to hyperammonemia (Cohn and Roth, 2004). Regardless of the cause, elevations of blood ammonia concentration above the normal physiological levels often lead to encephalopathies (Haberle, 2013). Interestingly, the clinicopathological features of acquired and congenital hyperammonemic encephalopathies such as coma, brain edema, and seizures (Summar et al., 2008; Haberle, 2013) are analogous to some of the features observed in CM patients (Idro et al., 2005). Moreover, experimental cerebral malaria (ECM) in murine models has also revealed metabolic changes that point toward parasite-induced perturbation of ammonia detoxification (Ghosh et al., 2012). These observations, coupled to the fact that *P. falciparum* generates large amounts of ammonia in the absence of intrinsic parasite detoxification mechanisms (Zeuthen et al., 2006) clearly suggests a potential role of parasite-derived ammonia in CM pathogenesis.

It was recently hypothesized that development of ALF in malaria patients may lead to accumulation of ammonia and other neurotoxins, which then enter the brain aided by blood brain barrier (BBB) breakdown and ultimately cause neurological damage and manifestation of CM (Martins and Daniel-Ribeiro, 2013). However, several studies indicates that parasite-induced liver dysfunction is not a common occurrence in *P. falciparum* malaria (Anand et al., 1992; Murthy et al., 1998; Nacher et al., 2001; Mazumder et al., 2002; Mohanty et al., 2004; Prommano et al., 2005; Kochar et al., 2010; Whitten et al., 2011). Therefore, it is conceivable that sequestered parasites in the brain vasculature known to produce large amounts of ammonia as a catabolic by-product, may disrupt normal brain ammonia metabolism leading to local accumulation of ammonia. This possibility has however not been considered as a pathogenic factor in CM. The aim of the current article is therefore to explore potential mechanisms by which *P. falciparum* may cause localized elevation of brain ammonia and subsequent neurotoxicity.

## Human brain ammonia uptake and metabolism

**Figure 1:**
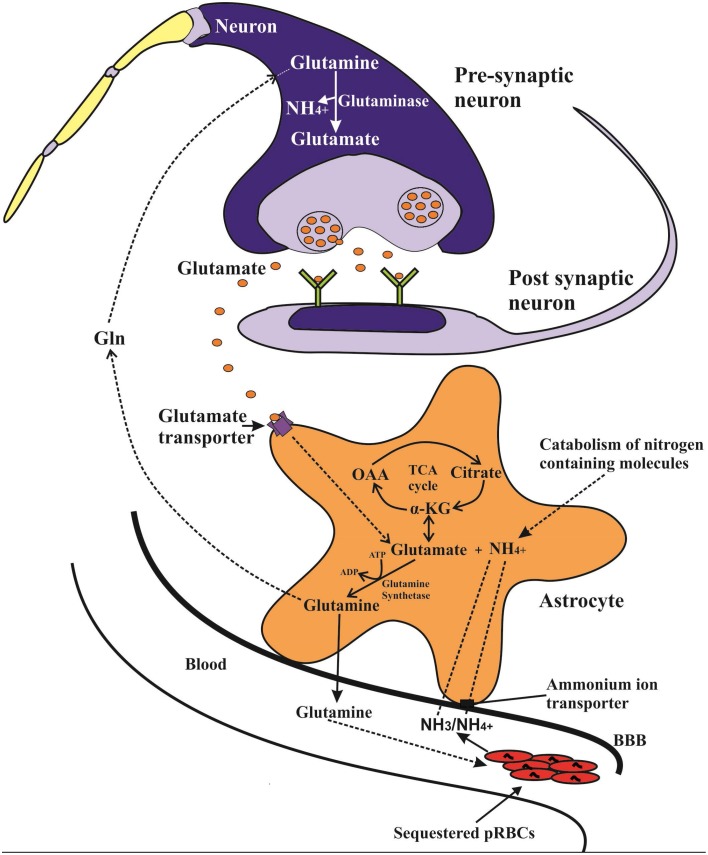
**Potential ammonia-glutamine vicious cycle in which intravascular malaria parasites catabolize glutamine, releasing ammonia, which is taken up into the astrocyte via transporters including NCCK1 and conjugated with glutamate (derived from α-ketoglutarate) to form glutamine in a glutamine synthetase (GS) catalyzed reaction**. The generated glutamine may then be released back to blood thus resupplying the parasite with the necessary glutamine. The GS catalyzed reaction also plays a key role in the glutamine-glutamate cycle, in which released synaptic glutamate is recycled back to the glutamatergic neurons in form of glutamine and converted by glutaminase to form glutamate neurotransmitter.

The human brain usually takes up ammonia from cerebral blood vessels using different transport mechanisms. The non-protonated NH_3_ form is easily taken up by simple diffusion and there is usually a positive linear correlation between arterial ammonia concentration and brain uptake (Cooper and Plum, 1987). The ammonium ion NH^+^_4_ on the other hand does not easily cross the BBB but is transported by carrier-mediated processes that utilize an array of potassium channels and transporters or by substituting other cations with similar hydrated radius (Ott and Larsen, 2004). Once in the astrocytes, ammonia combines with glutamate in an ATP-dependent reaction, catalyzed by glutamine synthetase (l-glutamate:ammonia ligase (ADP-forming; E.C.6.3.1.2) (GS) to form glutamine, which is then released into the blood stream (Cooper and Plum, 1987; Adeva et al., 2012) (Figure 1). Since the brain lacks effective urea cycle, this astrocyte GS catalyzed conversion of ammonia to glutamine and release into the blood stream is almost the only means to limit brain ammonia concentration (Cooper and Plum, 1987; Adeva et al., 2012). The astrocyte GS reaction also plays a role during acute systemic hyperammonemic conditions, where excess blood ammonia (NH_3_/ NH^+^_4_) is avidly taken up by the astrocytes and converted to glutamine, leading to increased brain and blood glutamine levels (Olde Damink et al., 2002; Brusilow et al., 2010).

Moreover, the astrocytic GS reaction is involved in glutamine-glutamate (GABA) cycle, which serves an important role in glutamatergic and GABAergic neurotransmission. In this cycle glutamate released from the glutamatergic neurons is taken up by the astrocytes and converted by GS reaction to glutamine, which is then transported back to neurons and converted to glutamate by neuronal phosphate-activated glutaminase with a concomitant release of ammonia (Zwingmann and Butterworth, 2005; Hertz, 2013).

## Can *P. falciparum* cause local elevation of brain ammonia levels?

The blood stage of *P. falciparum* degrades host hemoglobin to obtain free amino acids and imports some amino acids, particularly glutamine from the host serum (Elford et al., 1985; Goldberg, 2005). Although the parasite utilizes most of these free amino acids for protein biosynthesis, glutamine is usually deamidated and further deaminated and oxidized to 2-oxogluterate in order to maintain parasite's cellular levels of reduced NADPH + H^+^ (Werner et al., 2005). This deamidation and subsequent deamination reactions generates two molecules of ammonia per glutamine molecule. Thus, the malaria parasites require large amounts of glutamine and can produce substantial amounts of ammonia during the developmental life stages that require NADPH + H^+^. Indeed, an ammonia production rate of 0.8 fmol/h per parasite or 9 mmol/h per litre of packed infected erythrocytes has been demonstrated in the early trophozoite stage of *P. falciparum* (Zeuthen et al., 2006). Production of such amounts of ammonia may present a huge toxicity challenge to a parasite that lack ammonia-detoxifying machinery. However, studies suggest that the parasite may circumvent the toxic effects of ammonia by exporting it to the erythrocyte cytoplasm via *P. falciparum* aquaglyceroporin (PfAQP) and eventually into the extra-erythrocytic fluid (Zeuthen et al., 2006). Therefore, sequestration of a large number of metabolically active trophozoites and schizonts in a particular tissue vasculature may lead to release of massive amounts of ammonia into the blood stream.

In the human host, sequestration of PRBCs occur mainly in the brain microvasculature, with up to 10–20% of the total parasite biomass being estimated to be sequestered in the brain of some fatal CM cases (White et al., 2013). The sequestered PRBCs usually contain the mature trophozoite and schizonts (Silamut et al., 1999), which as already noted, takes up large amounts of glutamine from the extracellular fluid with concomitant increase in ammonia production (Elford et al., 1985; Zeuthen et al., 2006). The release of this parasite-derived ammonia in the human host most likely increases brain ammonia uptake, owing to reduced blood flow in congested microvessels and the fact that the brain ammonia uptake usually increases proportionally to the prevailing blood ammonia levels (Cooper and Plum, 1987). Subsequent detoxification of the increased brain ammonia via astrocytic GS reaction would in turn generate glutamine, which would then be released into the blood stream, as is the case during acute hyperammonemic conditions (Olde Damink et al., 2002). Interestingly, this would set up a vicious cycle whereby the sequestered parasites get rid of its toxic ammonia and at the same time sustains a continuous supply of glutamine by exploiting the host's astrocytic ammonia metabolic pathway (Figure 1). Such a cycle would enhance survival and rapid expansion of metabolically active sequestered parasite mass in the brain, thus sustaining and escalating ammonia production. Indeed malaria parasites are known to exploit or dysregulate other host's metabolic pathways to favor their survival and proliferation (Olszewski et al., 2009). However, the brain ammonia detoxification pathway capacity is limited and might be saturated by a sustained high rate of parasite-derived ammonia, thus leading to its accumulation and neurotoxicty. Moreover, but to a minor extent, an increased astrocytic glutamine output may also lead to increased glutamine uptake and subsequent release of ammonia by glutaminase-catalyzed reaction in the neurons. Therefore, *P. falciparum* particularly the sequestered parasites in the brain can create foci of increased ammonia production that may directly elevate brain ammonia, without the involvement of liver dysfunction.

Secondarily, other factors associated with *P. falciparum* infection may further contribute toward elevation of brain ammonia levels. Sequestered PRBCs, rosettes and reduced RBC deformability for example, may transiently reduce local microvascular perfusion leading to local tissue hypoxia. In such tissue regions, ammonia accumulation may be accelerated due to hypoxia-driven increase in amino acid catabolism (Cooper and Plum, 1987). Sequestered PRBCs in the brain may also create foci of enhanced immune cells activation and inflammatory responses (Porta et al., 1993; Patnaik et al., 1994; Medana et al., 2001). Activation of immune cells has been associated with increased uptake and utilization of glutamine to meet high energy demands required for effective immune functions (Curi et al., 1999). Glutamine catabolism by the activated immune cells may thus lead to enhanced ammonia production in the affected brain areas. Moreover, elevated reactive oxygen species (ROS) levels during their respiratory burst may compromise astrocytic ammonia detoxification events via inhibition of the the ROS sensitive GS (Brand et al., 1999), further aggravating the ammonia burden in the affected brain regions.

Hypoglycemia known to occur in up to one third of CM patients on admission (Idro et al., 2005) results in impaired energy metabolism and may also reduce ATP production. This would in turn exacerbate GS inhibition as a consequence of its reliance on ATP for enzymatic activity further contributing to ammonia accumulation. Fever and seizures, have also been shown to increase the rate of ammonia production in the vertebrate body (Cooper and Plum, 1987). Therefore, malaria induced fever and initial seizure episodes (mainly febrile) can contribute to heightened production of ammonia in the brain.

It is also possible that an initial slight to moderate increase in brain ammonia concentration by parasite's glutamine catabolic processes impairs the Krebs cycle, probably via inhibition of key metabolic enzymes like α-ketoglutarate (αKG) dehydrogenase (Cooper and Plum, 1987) and electron transport chain enzymes (Qureshi et al., 1998). Furthermore, the carbon needed to detoxify the excess parasite-derived ammonia depletes α-KG from cells, thereby additionally affecting the Krebs cycle (Ott et al., 2005). Pathophysiological concentrations of ammonia have also been shown to increase ROS production with resultant induction of mitochondrial permeability transition (MPT) in the astrocyte (Rama Rao et al., 2005). All these may ultimately reduce astrocyte ATP production thus setting up a vicious cycle in which ammonia impairs its own detoxification, leading to marked accumulation of ammonia and a further impairment of energy metabolism. Similarly, an initial parasite driven increase of ammonia can also decrease astrocyte expression of glutamate transporters (Chan et al., 2000) or increase ROS production (Rama Rao et al., 2005), further impairing ammonia brain metabolism.

In summary therefore, presence of a large number of metabolically active parasites in the brain can directly generate substantial amount of ammonia, as a metabolic byproduct, which may in turn overwhelm brain ammonia metabolism, leading to localized elevation of brain ammonia. The sequestration of PRBCs in the brain microvasculature is most likely the key factor that creates the large pool of parasites necessary to overwhelm the brain's ammonia detoxification capacity.

## Elevated brain ammonia hypothesis

**Figure 2:**
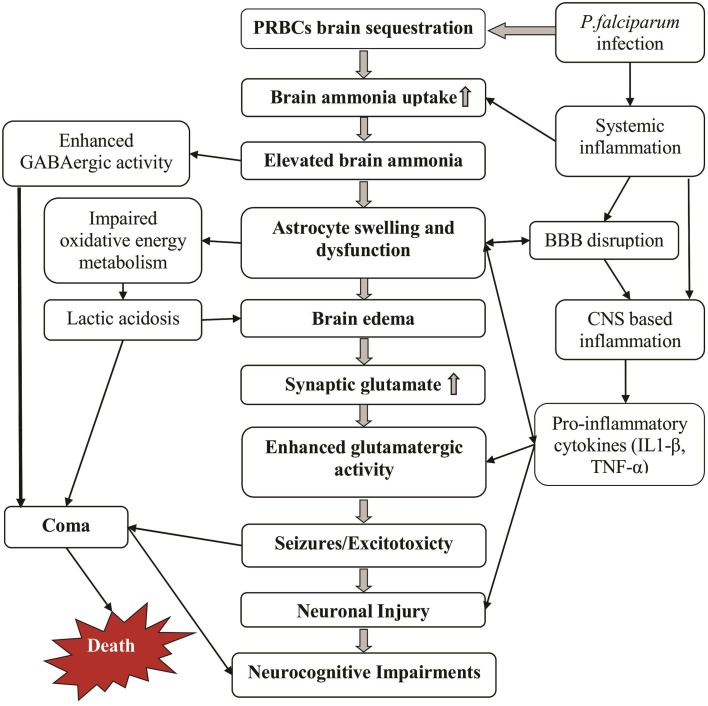
**Potential mechanisms by which cerebral malaria manifestations can develop**. Massive sequestration of parasites in brain microvasculature increases brain ammonia levels and thereby causing ammonia toxicity (middle and left columns). Some additional effects of the malaria infection including inflammation and disrupted BBB further enhance this toxicity (right column). The cause of death may be multifactorial including deep coma, edema and prolonged repetitive seizures.

In the human body ammonia exist at equilibrium as NH_3_(a weak base) and NH^+^_4_ (weak acid), with the latter having electrolytic conductance and ionic properties very similar to those of K^+^ ion (Bosoi and Rose, 2009). NH_3_ readily permeates membrane while the protonated NH^+^_4_ form crosses membranes via channels and substitution of K^+^ ion at its transporters (Nagaraja and Brookes, 1998). Elevated levels of ammonia alters brain function via direct as well as indirect mechanisms and has been considered to be a key factor in the pathogenesis of various metabolic encephalopathies (Butterworth et al., 1987; Butterworth, 1998; Bosoi and Rose, 2009; Haberle, 2013). We hypothesize that acute localized elevation of brain ammonia primarily by sequestered malaria parasites initiates a cascade of toxic effects that ultimately lead to the various CM clinical manifestations including brain edema, seizures, coma and neurological sequlae (Figure 2).

Brain edema and the associated raised intracranial pressure (ICP) and intracranial hypertension commonly observed in CM (Idro et al., 2005; Medana et al., 2011) may occur primarily due to ammonia-induced astrocyte swelling. This is because acute elevation of brain ammonia by the parasite and subsequent uptake into the brain would stimulate astrocytic GS catalyzed glutamine synthesis, leading to a rapid accumulation of glutamine. Intracellular accumulation of glutamine is known to cause astrocyte swelling via various mechanisms including increased osmotic stress (Norenberg et al., 1991; Butterworth, 1998), or enhanced oxidative/nitrative stress (ONS) and induction of MPT following uptake into the mitochondria (Albrecht and Norenberg, 2006). The physico-chemical similarities between NH^+^_4_ and K^+^ may also play a role in excess ammonia mediated atrocyte swelling as suggested by studies showing that ammonia activates Na-K-Cl cotransporter-1 (NKCC1) and oubain-activated signaling pathway (Rama Rao et al., 2005, 2014a; Jayakumar et al., 2011; Song and Du, 2014). Moreover, excess ammonia increases the levels of aquaporin 4 water channel (AQP4) in the brain (Pan et al., 2010; Bodega et al., 2012), which may play a role in astrocyte swelling and brain edema (Eefsen et al., 2010; Rama Rao et al., 2010b, 2014b). While these mechanisms are yet to be investigated in the pathogenesis of CM edema, a few recent studies points toward a potential role. Brain autopsy studies of CM patients with cereberal edema, for example found a trend toward increased AQP4 channels in the brain stem (Medana et al., 2011). Increased brain and blood glutamine levels have also been reported in ECM (Ghosh et al., 2012), lending support to our postulated ammonia-mediated genesis of cytotoxic edema in CM.

The brain endothelial cells also expresses NKCC1, whose activation increases Na^+^ and Cl^−^ transport into brain and has been implicated in the genesis of edema in stroke and experimental ischemic models (O'Donnell et al., 2004). Recent studies also indicate that ammonia activates the nuclear factor kappa B (NF-κB) pathway in brain endothelial cells, thereby contributing to astrocyte swelling and edema in ALF (Jayakumar et al., 2012, 2014). Therefore, it is possible that the widespread activation of brain endothelial cells (Combes et al., 2004; Idro et al., 2010) and up-regulation of NF-κB pathway by PRBCs (Tripathi et al., 2006, 2009) may partly be driven by the parasite-derived ammonia. The endothelial cell activation coupled with ammonia-mediated stimulation of BBB endothelial cell NKCC1 co-transporter activity, may in turn further contribute to the development and progression of brain swelling during CM.

Seizures and altered consciousness including deep coma are also commonly observed among CM patients, particularly children (Molyneux et al., 1989; Idro et al., 2010). We postulate that these manifestations are due to unique chemical properties of ammonia and its effects on key excitatory and inhibitory neurotransmitter systems at pathophysiological levels. A rapid increase in ammonia would not only result in intracellular alkalinization (increased intracellular pH) of astrocytes but also impairs extracellular K^+^ buffering capacity of the astrocytes (Marcaggi and Coles, 2001; Bosoi and Rose, 2009; Rangroo Thrane et al., 2013). The consequent rise in extracellular [K^+^] and overactivation of the Na^+^-K^+^-2Cl^−^cotransporter isoform 1 (NKCC1) in neurons in turn leads to selective impairment of cortical inhibitory GABAergic networks, neuronal disinhibition and seizures if ammonia concentration becomes very high (Rangroo Thrane et al., 2013).

Moreover, excess ammonia enhances excitatory glutamatergic neurotransmission via different mechanisms. Elevated ammonia for, example reduces astrocytic glutamate uptake and increases neuronal glutamate release, leading to elevated extracellular glutamate levels and enhanced excitatory glutamatergic activity (Raabe, 1987; Rose, 2002; Chan and Butterworth, 2003). Acute exposure of astrocytes to ammonia and consequent intracellular alkalinization also results in a calcium-dependent glutamate release (Rose et al., 2005). Collectively, these would increase extracellular glutamate thereby enhancing glutamatergic-mediated excitatory neurotransmission. Enhanced glutamatergic activity predispose the brain to seizures and excitotoxity (Meldrum, 1994; Stafstrom and Sasaki-Adams, 2003). Recent studies also suggest that prolonged exposure of astrocytes to extracellular glutamate down-regulates astrocytic inward rectifying potassium **(**Kir4.1) channels (Obara-Michlewska et al., 2011, 2014), a phenomenon which has been shown to be epileptogenic (Jansen et al., 2005).

In line with these observations, the seizures observed in CM may thus arise due ammonia-induced neuronal disinhibition and enhancement of glutamatergic activity. In support of these postulated mechanisms, biochemical changes indicative of increased glutamatergic activity have previously been reported in a murine CM model (Rae et al., 2004). The enhanced glutamatergic activity may however be transient since prolonged presence of increased glutamate in the synapse may cause downregulation of postsynaptic glutamate receptors, as an intrinsic protective response. Both N-methyl-D-aspartate (NMDA) and non-NMDA glutamate receptors for example are reduced in animal models of ALF, and this has been suggested to act as a counter mechanism against the detrimental effect of prolonged elevated extracellular levels of glutamate (Chan and Butterworth, 2003). Downregulation of glutamate mediated excitatory synaptic transmission, coupled with direct and indirect enhancement of GABAergic system by ammonia would depress brain activity, leading to coma (Basile, 2002). Indeed, an elevation of CNS tissue ammonia to 0.5 μmol/g, has been suggested to sufficiently disturb excitatory and inhibitory neurotransmission, leading to initiation of acute encephalopathy (Raabe, 1987).

Notably and of particular interest is that the coma in CM usually occurs rapidly often succeeding seizure onset and most patients recover quickly from the coma without much neurological impairments upon effective anti-malarial therapy (Molyneux et al., 1989; Idro et al., 2010). These observations fits well with our proposed patho-mechanisms and sequence of events i.e., elevated ammonia transiently enhances excitatory neurotransmission followed by compensatory measures that depresses brain activity ultimately leading to coma. Similarly, the rapid recovery from coma by most CM patients without much neurological impairment can be plausibly explained by our postulated mechanisms. A timely and effective anti-malarial therapy would basically reduce the quantity of sequestered ammonia-producing parasites, which in turn would promote restoration of normal brain ammonia metabolism, and return to normal neurotransmission.

Hypoglycaemia and lactic acidosis are also commonly observed among CM patients (Idro et al., 2005). While several factors such as reduced food intake, vomiting, hypoxia, heavy parasitemia, and systemic organ failures may lead to hypoglycemia and lactic acidosis, we hypothesize that ammonia-driven alteration of brain energy metabolism may play a major role. Ammonia disrupts the Krebs cycle via inhibition of alpha-ketoglutarate dehydrogenase (KGDH), leading to reduced mitochondrial production of ATP and reducing equivalents such as nicotinamide adenine dinucleotide (NADH) (Ott et al., 2005). Elevated ammonia also causes a metabolic shift toward glycolytic metabolism with concomitant increase in lactate formation (Kala and Hertz, 2005; Andersson et al., 2009). Increased lactate concentrations inhibit astrocytic glutamate uptake capacity (Andersson et al., 2009), which may further cripple the Krebs cycle through diminished anaplerotic replenishment of KGDH. The resultant reliance of the glycolytic pathway ATP production by the astrocytes in the wake of an increased need for ATP-dependent detoxification of excess ammonia would not only increase lactate and reduce pyruvate/lactate ratio but will also lead to rapid depletion of blood glucose. The lactic acidosis observed in CM may thus be largely due to ammonia-induced crippling of Krebs cycle and switch to the lactate producing glycolytic pathway. The observed hypoglycemia may be partly due to rapid depletion of glucose by the energetically inefficient glycolytic pathway, which generates only two molecules of ATP per glucose molecule. Such a consideration, may explain why restoration of normoglycemia in hypoglycemic CM patients, is not often associated with a change in the level of consciousness (Newton et al., 2000).

Increased CSF quinolinic acid and picolinic acid has been observed in children with CM but the exact cause is not well-understood (Dobbie et al., 2000; Medana et al., 2003). We hypothesize that the effects of elevated brain ammonia on tryptophan uptake and metabolism play a key role. Generally, free tryptophan is metabolized via the kynurenine pathway into a series of bioactive metabolites including kynurenine, kynurenic acid, 3-Hydroxykynurenine, 3-Hydroxyanthranilic acid, picolinic acid, anthranilic acid, xanthurenic acid, and quinolinic acid (Stone and Darlington, 2002; Stone et al., 2013). Among these metabolites, quinolinic acid is an excitotoxic N-methyl-D-aspartate (NMDA) glutamate receptor agonist, while kynurenic acid is a neuroprotective glutamate receptor antagonist (Stone and Perkins, 1981; Stone, 2001; Schwarcz and Pellicciari, 2002; Guillemin, 2012). Quinolinic acid is also a pro-oxidant, an immunomodulator and promotes the formation of hyperphosphorylated tau proteins in the nervous system (Guillemin et al., 2003; Rahman et al., 2009). Notably, the synthesis of the neuroprotectant kynurenic acid in the brain occur mainly within the astrocytes, while the synthesis of 3-hydroxykynurenine and further downstream metabolites including quinolinic acid occur in microglia (Stone et al., 2013). Excess ammonia is known to increase brain tryptophan uptake even in absence of any liver function derangement (Bachmann and Colombo, 1983). It also inhibits kynurenic acid synthesis from kynurenine in a dose-dependent manner (Saran et al., 1998) and increases cerebral blood flow (Aggarwal et al., 1994; Jalan et al., 2004b). By increasing cerebral blood flow as CM progresses, elevated ammonia may thus increase tryptophan uptake from the blood. However, subsequent metabolism will be skewed in favor of increased level of microglia-derived quinolinic acid and less of neuroprotectant kynurenic acid since elevated ammonia also reduces kynurenic acid synthesis (Saran et al., 1998). The increased quinolinic acid would in turn contribute to altered NMDA receptor activity and excitotoxity (Stone and Perkins, 1981; Guillemin, 2012) as well as increased CNS proinflammatory cytokine production and oxidative stress (Guillemin et al., 2003).

Systemic inflammatory response is common during *P. falciparum* malaria infections, leading to increased blood concentration of both pro-inflammatory and anti-inflammatory cytokines. Among some patients who develop CM, various blood pro-inflammatory cytokines have been reported to increase including interleukin 1 beta (IL-1β), IL-6, and tumor necrosis factor alpha (TNF-α) (Kwiatkowski et al., 1990; Ringwald et al., 1991; Brown et al., 1999; Wenisch et al., 1999; Akanmori et al., 2000; Lyke et al., 2004). Therefore, a key role of inflammation in CM pathogenesis and pathology cannot be overlooked. We hypothesize that parasite-elicited systemic inflammation potentiates the proposed elevation of brain ammonia and subsequent neurotoxicity. This may include potentiation of brain ammonia uptake (Duchini et al., 1996) as well as the untoward effects of excess ammonia on neurocognitive functions (Shawcross et al., 2004; Marini and Broussard, 2006), astrocyte osmotic balance (Oh et al., 1992; Rama Rao et al., 2010a) and synaptic neurotransmission (Casamenti et al., 1999; Hu et al., 2000; Medana et al., 2001). The systemic inflammation may also synergize ammonia-induced pathogenic alteration of cerebral blood flow (CBF) and increased ICP as previously observed both in human and animal studies of hyperammonemia (Jalan et al., 2004b; Pedersen et al., 2007). Moreover, inflammatory cytokines particularly TNF-α promotes sequestration of PRBCs (Idro et al., 2010), the primary event that leads to elevation of brain ammonia according to our hypothesis.

Potentially also, elevated brain ammonia may elicit CNS based inflammatory response via direct activation of microglia and astrocytes. This is because CNS based cells particularly astrocytes, microglia, and oligodendrocytes are known to initiate inflammation in response to local noxious stimuli (Lucas et al., 2006) and high concentrations of ammonia has been shown to be a potent inflammatory stimuli. In one *in vitro* study, for example, ammonia was reported to directly stimulate secretion of TNF-α and IL-8 by immortalized human microglia (CHME-5) and astroglioma (GL-15) cells even in the absence of any other stimulus (Atanassov et al., 1995). Such a possibility of ammonia induced CNS inflammatory response, may explain the observed widespread activation of microglial throughout the brain in white and gray matter and far beyond areas of petechial bleedings or sequestered PRBCs foci during CM (Schluesener et al., 1998). It may also explain the induction and CNS production of the pro-inflammatory cytokines that has been observed in children with CM (Brown et al., 1999; John et al., 2008). Moreover, it may explain the therapeutic failure of drugs such as pentoxifylline (Idro et al., 2010), since such drugs may not cross the BBB to inhibit activated microglia.

Apoptosis of neurons, endothelial cell and astrocytes has been suggested to play a role in the genesis of symptoms and signs of experimental animal CM (Wiese et al., 2006; Lackner et al., 2007). Evidence also suggest that *P. falciparum* induces apoptosis of neurons, brain endothelial cell and astrocytes and might play a role in the pathogenesis of human CM pathology (Pino et al., 2003; Toure et al., 2008; Idro et al., 2010; N'dilimabaka et al., 2014). However, the cause and exact mechanisms underlying induction of apoptosis in these cells is not fully understood. Elevated brain ammonia might play a role. In one study, exposure of C6 glioma cells to 5 or 10 mM ammonia for 96 h induced apoptosis in 50% of the cells, via activation of Protein kinase C (PKC) and nuclear transcription factor kappa B (NFκB) pathways (Buzanska et al., 2000). In another study, ammonia was shown to induce apoptosis of rat hippocampal neurons via calcineurin-mediated BAD Ser155 dephosphorylation (Yang et al., 2004). Ammonia was also reported to induce apoptosis in primary cortical neurons, possibly via reduced phosphorylation and activation of protein kinase B (PKB or Akt), a key component in the activation of cellular pro-survival pathways (Klejman et al., 2005). Over activation of NMDA receptors which can be caused by elevated ammonia, has also been shown to induce cleavage and activation of pro-apoptotic caspases (Tenneti et al., 1998).

Interestingly, similar mechanisms seem to mediate the apoptosis in experimental animal CM and *P. falciparum in vitro* models. Activation of pro-apototic caspase 3, for example, was found to play a key role in neuronal and astrocytic apoptosis in experimental murine CM (Pino et al., 2003; Potter et al., 2006; Lackner et al., 2007). Recently also, reduced activation of Akt and decreased inhibition of the glycogen kinase synthase beta (GSK3β) was demonstrated in the brains of mice infected with *Plasmodium berghei* ANKA (Dai et al., 2012). Our postulated parasite-driven elevated brain ammonia might thus activate pro-apoptotic pathways and impair of pro-survival pathways, leading to apoptosis of neurons, astrocytes, and endothelial cells, thereby contributing to acute and long-term neuropathology.

Elevated brain ammonia can lead to long-term neurocognitive sequlae observed among CM survivors via various pathomechanisms. First, the widespread activation of microglia by elevated ammonia might predispose neurons to oxidative burst and lysosomal damage (Schluesener et al., 1998). Secondly, the overactivation of NMDA receptors by increased extracellular glutamate acting in synergy with high brain quinolinic acid, IL-1ß, and TNF-α concentration can lead to excitotoxic damage of NMDA receptor containing neurons and/or “kindling.” Such a mechanism may be particularly important in the development of long-term learning impairment and increased susceptibility to epilepsy later on in life among peadiatric CM survivors, as indicated by studies in young rats (Stafstrom and Sasaki-Adams, 2003). Thirdly, the derangement of energy metabolism within the brain as a result of elevated ammonia-mediated Krebs cycle inhibition can cause brain cellular damage or death, possibly via enhanced cerebral proteolysis as proposed by Ott et al. (2005). Fourth, the ammonia induced astrocyte swelling and ROS production, α-KDH inhibition and pro-oxidant kynurenines production may predispose the neurons to enhanced damaging effects of oxidative stress. Additionally and perhaps most important is that neurcognitive sequlae may develop due to ammonia induced apoptotic death of brain cells (Buzanska et al., 2000; Yang et al., 2003, 2004). Any of these mechanisms acting singly or cooperatively could lead to neuronal damage, death or persistent disturbed synaptic processes, which may in turn manifest as long-term neurocognitive impairments, following a CM episode.

Consideration of parasite-driven brain ammonia elevation as a central pathogenic factor can therefore account for the genesis of the various manifestations observed in CM patients. It also allows for the integration of various patho-mechanisms into a clear sequence of events during the genesis and progression of CM. Although there are several ethical and practical challenges to testing of the hypothesized mechanisms in humans with CM, a number of approaches are possible. CSF ammonia, glutamine, aspartate, and alanine levels can be measured in well-defined groups of CM patients. Post mortem analysis of ammonia metabolizing enzymes and level of ammonia metabolism related amino acids like glutamate, glutamine, and aspartate could also be done in brain tissues obtained from fatal cases of cerebral malaria. The outcome of clinical interventions that limits either blood, brain ammonia levels, or prevent ammonia-induced brain dysfunctions may also provide an indirect additional approach.

Murine models of CM can also provide an alternative approach to test the current hypothesis and its predictions with the added advantage of allowing experimental manipulations. Blood, CSF, and brain ammonia levels can be studied in both the fatal and resolving mouse models of CM. Similarly, pre-mortem and post-mortem studies of key enzymes involved in ammonia metabolism including glutamine synthetase, glutamate dehydrogenase, and glutaminase can provide data to evaluate this hypothesis.

## Therapeutic implications

If elevated brain ammonia plays a central role in CM pathogenesis, then interventions that reduces ammonia formation or toxicity could potential improve CM treatment outcome. Protein intake restriction during cerebral malaria and promotion of anabolism by rapid administration of intravenous dextrose solution could therefore reduce ammonia formation and toxicity. Similarly, aggressive control of factors such as fever and seizures, which enhances ammonia formation, may be beneficial.

Chemical compounds that act as metabolic “sinks” for ammonia can also be explored as adjuncts in the management of CM. These include compounds such as benzoate, sodium phenylacetate, and calcium phenylbutyrate. These agents are currently used in the management of various hyperammonemic disorders. Hypothermia and other interventions aimed at mitigating ammonia induced brain dysfunction and damage may also be tested as potential adjunct therapies in the management of CM. Hypothermia has been found to reduce ICP in patients with ALF (Jalan et al., 1999, 2004a). It would be therefore interesting to test the efficacy of mild hypothermia in reducing CM-induced brain edema and raised ICP. More recently, several experimental agents including NMDA receptor antagonists, drugs antagonizing endogenous ammonia-activated ouabain-like compounds, Na-K-Cl cotransporter-1 (NKCC1) inhibitors, and L-histidine have shown therapeutic potential in ALF animal models (Rama Rao et al., 2010c; Jayakumar et al., 2011; Cauli et al., 2013; Song and Du, 2014). These agents may also represent novel and potentially relevant experimental adjunctive therapies in CM.

## Conclusion

The similar clinicopathological features of CM and hyperammonemic encephalopathies as well as the available evidence suggesting that *P. falciparum* can produce substantial amount of ammonia warrants the consideration of parasite-derived ammonia as a potential key factor in the pathogenesis of cerebral malaria. Predictable consequences of *P. falciparum*-induced elevation of brain ammonia can account for most of clinicopathology of CM and therefore provide hints of potential adjunctive therapies. Targeting of the parasite's ammonia production and transport via PfAQP blockade could also be a novel therapeutic strategy.

### Conflict of interest statement

The authors declare that the research was conducted in the absence of any commercial or financial relationships that could be construed as a potential conflict of interest.
